# Safety and tolerability of long-term treatment with darolutamide in patients with metastatic castration-resistant prostate cancer

**DOI:** 10.1038/s41391-023-00740-9

**Published:** 2023-10-26

**Authors:** Robert Hugh Jones, Karim Fizazi, Nicholas D. James, Teuvo L. Tammela, Nobuaki Matsubara, Frank Priou, Philippe Beuzeboc, Thierry Lesimple, Petri Bono, Vesa Kataja, Jorge A. Garcia, Andrew Protheroe, Neal Shore, John Aspegren, Heikki Joensuu, Iris Kuss, Sabine Fiala-Buskies, Egils Vjaters

**Affiliations:** 1 Cardiff University and Velindre Cancer Centre, Cardiff, UK; 2 Institut Gustave Roussy, University of Paris Saclay, Villejuif, France; 3 The Institute of Cancer Research, London, UK; 4 Tampere University Hospital, Tampere, Finland; 5 National Cancer Center Hospital East, Chiba, Japan; 6 Centre Hospitalier Départemental Vendée, La Roche-Sur-Yon, France; 7 Curie Institute, Paris, France; 8 Centre Eugène Marquis, Rennes, France; 9 Terveystalo Finland and University of Helsinki, Helsinki, Finland; 10 Kuopio University Hospital, Kuopio, Finland; 11 University Hospitals Seidman Cancer Center, Case Comprehensive Cancer Center, Cleveland, OH USA; 12 Oxford University Hospitals NHS Foundation Trust, Oxford, UK; 13 Carolina Urologic Research Center, Atlantic Urology Clinics, Myrtle Beach, SC USA; 14 Orion Corporation, Espoo, Finland; 15 Bayer AG, Berlin, Germany; 16 Bayer AG, Wuppertal, Germany; 17 P. Stradins Clinical University Hospital, Riga, Latvia

**Keywords:** Prostate cancer, Cancer therapy

## Abstract

**Background:**

In patients with metastatic castration-resistant prostate cancer, darolutamide was well tolerated for 25 months, but minimal long-term safety data are available.

**Methods:**

Treatment-emergent adverse events (TEAEs) for patients receiving darolutamide for a median of 38 months (*n* = 13) are described in this pooled analysis of individual patient data from phase 1/2 studies.

**Results:**

All patients reported TEAEs (mostly grade 1/2). The most common TEAEs were diarrhea, abdominal pain, and nausea. Serious TEAEs were reported in six patients (none related to darolutamide). All treatment-related TEAEs (*n* = 5) were grade 1.

**Conclusions:**

Long-term darolutamide treatment was well tolerated; no new safety signals observed.

**Tweetable abstract:**

In patients with mCRPC, long-term darolutamide treatment was well tolerated and no new safety signals were observed. These findings are consistent with previous reports, demonstrating a favorable safety and tolerability profile of darolutamide.

Darolutamide is a structurally distinct and highly potent androgen receptor inhibitor (ARi) that demonstrated strong efficacy and a consistently favorable safety and tolerability profile in the phase 3 ARAMIS and ARASENS studies in patients with nonmetastatic castration-resistant prostate cancer (nmCRPC) and patients with metastatic hormone-sensitive prostate cancer (mHSPC) [1, 2]. Darolutamide in combination with androgen-deprivation therapy (ADT) or ADT and docetaxel, respectively, reduced the risk of death by more than 30% compared with placebo [1, 2]. In ARAMIS, most treatment-emergent adverse events (TEAEs) commonly associated with ARi therapy showed ≤2% difference between darolutamide and placebo, and discontinuation rates due to TEAEs were low and similar to placebo (8.9% vs 8.7%) [1]. The overall incidence of TEAEs was also similar between darolutamide and placebo in ARASENS, with the highest incidences of TEAEs occurring during the overlapping docetaxel treatment period [2].

Among patients with metastatic castration-resistant prostate cancer (mCRPC), darolutamide was well tolerated for up to 25 months in the previously reported ARADES, ARAFOR, and Japanese phase 1/2 studies [3–5]. ARADES was a phase 1 dose-escalation/phase 2 randomized dose-expansion study (NCT01317641/NCT01429064) of 136 patients in whom darolutamide was administered twice daily (BID) at doses of 200, 300, or 700 mg [3]. The ARAFOR study (NCT01784757) was a food-effect study that enrolled 30 patients who received darolutamide 600 mg BID [4]. A Japanese phase 1 study (NCT02363855) included nine patients who received darolutamide 300 mg or 600 mg BID [5]. Minimal safety data are available from extended treatment with darolutamide in this population. Thus, we performed a pooled analysis of individual patient data including mCRPC patients who were treated with darolutamide for >2 years. Patients were assessed every 3 months to evaluate the safety and tolerability of long-term darolutamide.

A total of 13 patients received darolutamide for >2 years, with seven patients receiving 2 to 4 years of treatment and six patients receiving more than 4 years of treatment. The median (range) age of patients was 68 (55–81) years, 12 patients were White, and one patient was Asian. Patients were treated in France (*n* = 4), the United Kingdom (*n* = 3), Latvia (*n* = 3), Finland (*n* = 2), and Japan (*n* = 1). Most patients had normal renal and hepatic function (77%), Eastern Cooperative Oncology Group performance status of 0 (92%), and Gleason score of seven or higher (69%). The median (range) time from initial diagnosis until first darolutamide dose was 32.4 (9.7–191.0) months, and median (range) baseline prostate-specific antigen level before initiating darolutamide was 18.1 (4.6–53.6) μg/L. All patients received endocrine therapy before darolutamide treatment (bicalutamide, 77%; triptorelin acetate, 46%; goserelin/goserelin acetate, 31%; leuprorelin acetate, 23%; cyproterone acetate, 23%; degarelix/degarelix acetate, 15%; abiraterone, 8%). No patient received prior chemotherapy. The median (range) treatment duration of darolutamide was 38.1 (24.2–90.0) months for all 13 patients receiving >2 years of treatment and 62.6 (48.6–90.0) months for those receiving >4 years of treatment. One patient completed ARAFOR and entered the darolutamide rollover study (NCT04464226).

TEAEs were reported by all 13 patients, with worst grade 1 or 2 events in seven patients and worst grade 3 events in six patients; no grade 4 or 5 events were reported (Table 1). No grade 3 events occurred in more than one patient and none were considered related to darolutamide. Grade 3 TEAEs included rectal adenocarcinoma and presyncope in a patient treated for >2 and ≤4 years and one event each of nausea, hepatic cirrhosis, pyelonephritis, accident, femoral neck fracture, spinal compression fracture, paraneoplastic syndrome, hematuria, lung disorder, and pulmonary embolism in five patients treated for >4 years. Serious TEAEs were reported in six patients and none were considered related to darolutamide. One patient treated for >2 years developed a new locally advanced rectal adenocarcinoma that was not considered related to darolutamide but led to treatment discontinuation. Five patients reported TEAEs considered related to darolutamide (tinnitus, abdominal distention, diarrhea, dyspepsia, fatigue, gynecomastia, and solar dermatitis) and all were grade 1 in severity. No treatment-related TEAEs led to discontinuation of darolutamide. The most common TEAEs were diarrhea, abdominal pain, and nausea (Table 1). During long-term darolutamide therapy, few patients experienced TEAEs commonly associated with ARi therapy (fatigue, *n* = 3; falls, including accidents, *n* = 3; bone fractures, *n* = 2; hypertension, *n* = 2; rash, *n* = 1).

**Table 1:** Treatment-emergent adverse events during long-term darolutamide treatment.

Treatment-emergent adverse events (TEAEs),^a^ *n* (%)	Total: Darolutamide >2 years (*n* = 13)	Darolutamide >2 and ≤4 years (*n* = 7)	Darolutamide >4 years (*n* = 6)
Any TEAE	13 (100)	7 (100)	6 (100)
Worst grade			
1 or 2	7 (54)	6 (86)	1 (17)
3	6 (46)	1 (14)	5 (83)
Serious TEAE	6 (46)	2 (29)	4 (67)
TEAE leading to discontinuation of darolutamide	1 (8)	1 (14)	0
Any drug-related TEAE	5 (38)	3 (43)	2 (33)
Worst grade			
1 or 2	5 (38)	3 (43)	2 (33)
3	0	0	0
Serious drug-related TEAE	0	0	0
Drug-related TEAE leading to discontinuation of darolutamide	0	0	0
Most common TEAEs (occurring in ≥3 patients)^b^			
Diarrhea	5 (38)	2 (29)	3 (50)
Abdominal pain	4 (31)	2 (29)	2 (33)
Nausea	4 (31)	2 (29)	2 (33)
Arthralgia	3 (23)	1 (14)	2 (33)
Fatigue	3 (23)	2 (29)	1 (17)
Hematuria	3 (23)	1 (14)	2 (33)
Influenza	3 (23)	1 (14)	2 (33)

^a^Treatment-emergent adverse events were assessed according to National Cancer Institute Common Terminology Criteria for Adverse Events (CTCAE) version 4.03.

^b^All TEAEs were grade 1 or 2 except 1 event each of grade 3 nausea and grade 3 hematuria.

The favorable safety and tolerability profile of long-term darolutamide treatment in patients with mCRPC are consistent with that previously reported from the early phase studies and in patients with nmCRPC and mHSPC [1–5]. In addition, extended treatment with darolutamide in the open-label phase of the ARAMIS trial (median treatment duration 25.8 months) showed minimal increase in the incidences of TEAEs from the darolutamide double-blind period (18.5 months) that disappeared when adjusted for longer exposure, and no new safety signals were observed [6, 7]. The randomized, crossover, phase 2 ODENZA trial compared darolutamide and enzalutamide in 200 patients with mCRPC for treatment preference and cognitive outcomes [8]. Darolutamide was preferred by 48.5% of patients versus 40.0% for enzalutamide (*P* = 0.92). A clinically meaningful benefit in episodic memory and less fatigue was observed in patients receiving darolutamide compared with those receiving enzalutamide. Matching-adjusted indirect comparisons and meta-analyses have shown a lower risk of adverse events and similar efficacy with darolutamide versus apalutamide and enzalutamide [9, 10].

In conclusion, long-term treatment with darolutamide in this small group of patients with mCRPC was well tolerated. These findings provide valuable information for patients and clinicians in selecting treatment for prostate cancer.

## Data Availability

Availability of the data underlying this publication will be determined according to Bayer’s commitment to the EFPIA/PhRMA “Principles for responsible clinical trial data sharing.” This pertains to scope, timepoint and process of data access. As such, Bayer commits to sharing upon request from qualified scientific and medical researchers patient-level clinical trial data, study-level clinical trial data, and protocols from clinical trials in patients for medicines and indications approved in the United States (US) and European Union (EU) as necessary for conducting legitimate research. This applies to data on new medicines and indications that have been approved by the EU and US regulatory agencies on or after January 1, 2014. Interested researchers can use www.vivli.org to request access to anonymized patient-level data and supporting documents from clinical studies to conduct further research that can help advance medical science or improve patient care. Information on the Bayer criteria for listing studies and other relevant information is provided in the member section of the portal. Data access will be granted to anonymized patient-level data, protocols and clinical study reports after approval by an independent scientific review panel. Bayer is not involved in the decisions made by the independent review panel. Bayer will take all necessary measures to ensure that patient privacy is safeguarded.
